# Enhancement of Diabetic Retinopathy Prognostication Using Deep Learning, CLAHE, and ESRGAN

**DOI:** 10.3390/diagnostics13142375

**Published:** 2023-07-14

**Authors:** Ghadah Alwakid, Walaa Gouda, Mamoona Humayun

**Affiliations:** 1 Department of Computer Science, College of Computer and Information Sciences, Jouf University, Sakakah 72341, Al Jouf, Saudi Arabia; gnalwakid@ju.edu.sa; 2 Department of Electrical Engineering, Faculty of Engineering at Shoubra, Benha University, Cairo 11672, Egypt; walaa.gouda@feng.bu.edu.eg; 3 Department of Information Systems, College of Computer and Information Sciences, Jouf University, Sakakah 72341, Al Jouf, Saudi Arabia

**Keywords:** blindness, diabetic retinopathy, Deep Learning, APTOS, transfer learning

## Abstract

One of the primary causes of blindness in the diabetic population is diabetic retinopathy (DR). Many people could have their sight saved if only DR were detected and treated in time. Numerous Deep Learning (DL)-based methods have been presented to improve human analysis. Using a DL model with three scenarios, this research classified DR and its severity stages from fundus images using the “APTOS 2019 Blindness Detection” dataset. Following the adoption of the DL model, augmentation methods were implemented to generate a balanced dataset with consistent input parameters across all test scenarios. As a last step in the categorization process, the DenseNet-121 model was employed. Several methods, including Enhanced Super-resolution Generative Adversarial Networks (ESRGAN), Histogram Equalization (HIST), and Contrast Limited Adaptive HIST (CLAHE), have been used to enhance image quality in a variety of contexts. The suggested model detected the DR across all five APTOS 2019 grading process phases with the highest test accuracy of 98.36%, top-2 accuracy of 100%, and top-3 accuracy of 100%. Further evaluation criteria (precision, recall, and F1-score) for gauging the efficacy of the proposed model were established with the help of APTOS 2019. Furthermore, comparing CLAHE + ESRGAN against both state-of-the-art technology and other recommended methods, it was found that its use was more effective in DR classification.

## 1. Introduction

**Figure 1:**
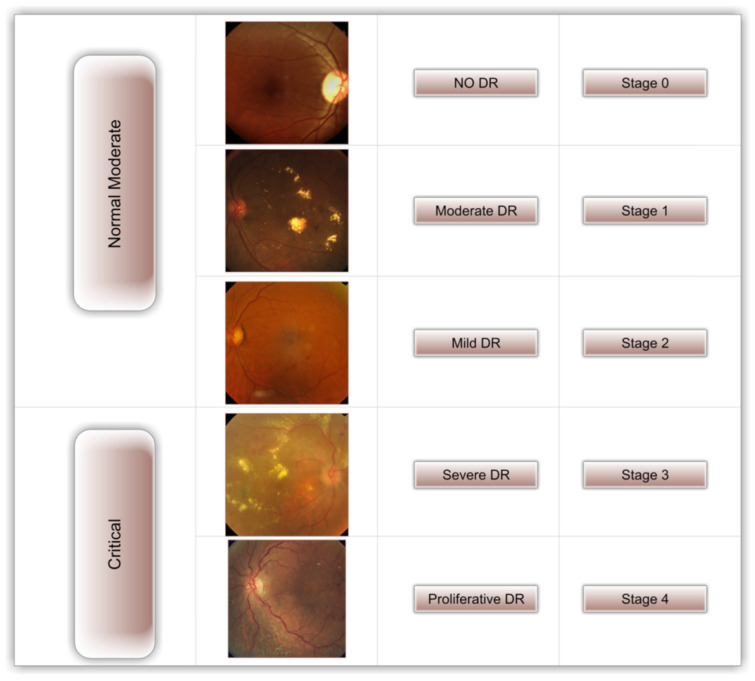
Listed in order of increasing severity, the five stages of DR.

Diabetes can lead to several serious complications, including DR, visual loss, cardiovascular disease, kidney disease, and strokes. DR occurs when excess glucose levels inflame and leak into the retinal vessels [1,2,3]. Lesions show up as blotches of blood and fluids on the retina. Primarily, a DR diagnostic will involve looking for red, brilliant lesions. The red lesions involve microaneurysms (MA) and hemorrhage (HM), while the bright lesions involve soft and hard exudates (EX). The smaller, dark red dots are MA, while the more prominent spots are HM. Injuries to nerve fibers cause soft EX to look like yellowish-white, fluffy specks, while nerve damage causes hard EX to appear as definite yellow spots [4,5]. Figure 1 depicts the five distinct stages of DR (no DR, mild DR, moderate DR, severe DR, and proliferative DR) [6,7]. When DR progresses to its most severe stage, a person’s vision may be lost totally. Early detection of DR can reduce the likelihood of permanent vision loss [4,8].

Experts in the field are needed to diagnose DR manually, but even the most seasoned ophthalmologists struggle with interpersonal and inter-observer inconsistency; however, early detection of DR is crucial for preventing blindness [9,10]. As a result, numerous Machine Learning (ML) and Deep Learning (DL) algorithms for automatic DR detection have been developed by academics throughout the past decade. Though reliable DR detection via image analysis via DL has come a long way, there is still much room for improvement. Several studies on DR detection have utilized single-stage training for the entire process [11,12,13,14].

To help ophthalmologists with DR assessments, we aimed to create a fast, highly autonomous, DL-based DR classification. When DR is recognized and treated soon after the first signs of the condition arise, it can be avoided. We used the freely available APTOS dataset [15] to train a model with cutting-edge image preprocessing techniques and the DenseNet-121 [16] model for diagnosis.

Within this section, we focus on the novel aspects of our research.

1. The primary contribution of this study is that it employs the contrast-limited adaptive HIST (CLAHE) [17] filtering technique, HIST [18], and ESRGAN [19] to produce superior images for the APTOS collection;
2. The suggested system’s sustainability is assessed through comparative research using a variety of metrics such as accuracy, precision, confusion matrix, recall, top-n accuracy, and the F1-score;
3. The APTOS data collection serves as the basis for training the DenseNet-121 pre-trained model;
4. Using the augmentation method, We ensured an even amount of information in the APTOS dataset;
5. Overfitting occurs less frequently, and the suggested method’s underlying trustworthiness is enhanced by using a training technique that accommodates several different training strategies (e.g., learning rate, batch size, data augmentation, and validation patience).

This study presents three different scenarios: Scenario I, in which CLAHE and ESRGAN are used together to optimize the DR stage enhancement; scenario II, in which CLAHE is used first and then HIST and ESRGAN, respectively; and scenario III, in which HIST is used first and then CLAHE and ESRGAN are applied to the images. Furthermore, we used DenseNet-121 to train the weights for each scenario, utilizing images from the APTOS dataset to assess the models’ outputs. Oversampling through augmentation methods is essential because of the class imbalance in the dataset. The rest of the paper will be written following this outline. Section 2 provides a background on the DR, while Section 3 lays out a plan for performing the study. Section 4 presents and discusses the findings. Final thoughts and suggestions for further research are presented in Section 5.

## 2. Related Work

Manually detecting DR in images was fraught with complications. A lack of capability (qualified ophthalmologists) and expensive examinations present obstacles for many people in poor nations. Automatic processing methods have been developed to increase access to precise and prompt assessment and treatment for blindness, as early detection is crucial in the struggle against the disease. ML models fed images of the retinal fundus have recently achieved high accuracy in DR categorization [2,20]. While the end result of using ML algorithms was promising, extracting the features using methods for image processing takes more work. In computer vision and bioinformatics, DL models have recently demonstrated increased effectiveness. So, many studies have been developed using DL models to identify DR in fundus retinal images. Some researchers have adopted a transfer learning strategy to deal with the limited space available in DR Datasets.

Gundluru et al. [21] created a DL model with PCA for dimensionality reduction and Harris Hawks optimization for better feature extraction and categorization. Yasin et al. [22] propose a three-stage procedure. After preprocessing retinal pictures, the hybrid Inception-ResNet architecture classified the image development stages. Finally, DR severity is low, moderate, severe, or proliferative. Farag et al. [23] offer an autonomous DL severity detection approach employing a single-Color Fundus picture (CFP). DenseNet169 embeds visuals. CBAM enhances discrimination. Finally, cross-entropy loss trains the APTOS dataset model.

Using transfer learning and pre-trained models (NASNetLarge, EfficientNetB4, Xception, EfficientNetB5, and InceptionResNetV2), Liu et al. [24] predicted DR on the EyePACS dataset. The DR was successfully categorized using an improved cross-entropy loss function and three hybrid model structures, achieving an accuracy of 86.34%. For DR recognition in fundus pictures, Sheikh et al. [25] used a combination of four transfer learning algorithms: VGG16, ResNet50, InceptionV3, and DenseNet-121. With 90% sensitivity and 87% specificity, DenseNet-121 outperformed competing models in predictive accuracy. 

On top of a pre-trained InceptionResNetv2, Gangwar and Rav [26] developed a unique convolutional neural network (CNN) module. Two datasets, Messidor-1 and APTOS 2019, were used to hone those models. The Messidor-1 dataset earned 72.33 percent accuracy, while the APTOS 2019 dataset scored 82.18 percent accuracy during testing.

Omneya Attallah [27] proposes a powerful and automated CAD tool built on the back of GW and a number of other DL models. Saranya et al. [28] used red lesions in retinal pictures to construct an automated model for early DR detection. Preprocessing removes noise, improves local contrast, and uses the UNet architecture to semantically partition red lesions. Medical segmentation requires pixel-level class labeling, which U-Net supports with Advanced CNN. The model was tested using four publicly available datasets: IDRiD, DIARETDB1, MESSIDOR, and STARE. Using the IDRID dataset, the suggested identification system had 99% specificity, 89% sensitivity, and 95.65% accuracy. Using the MESSIDOR dataset, the DR severity classification system had 93.8% specificity, 92.3% sensitivity, and 94% accuracy. 

Raiaan et al. [29] established a new dataset by merging images from the APTOS, Messidor2, and IDRiD datasets. Image preprocessing and geometric, photometric, and elastic deformation augmentation methods are applied to all images in the dataset. RetNet-10 is a base model containing three blocks of convolutional layers and maxpool layers and a categorical cross-entropy loss function to classify DR stages. The RetNet-10 model had a high testing accuracy of 98.65%.

Xu et al. [30] suggested a DL model that achieved 94.5 percent accuracy in automated DR classification. They used several different augmentations to deal with the overfitting issue introduced by the small dataset. By first collecting spatial features from the four TL and then integrating these features using the Fast Walsh Hadamard Transform, Omneya Attallah [31] can identify meaningful features. The data they obtained had an accuracy of 93.2%. A segment-based learning system for DR prediction was reported by Math et al. [32]. The area under the ROC curve was 0.963 when they utilized a pre-trained CNN to estimate DR at the segment level and classify all segment levels. On the EyePACS dataset, Kaushik et al.’s [33] stacked model of 3 CNN models achieved 97.92% binary classification and 87.45% multi-class classification. They segmented and localized blood vessels, microaneurysms, hemorrhages, exudates, and other lesions in addition to image-level grading for DR categorization. Medical DR detection algorithms were investigated by Khalifa et al. [34], who used deep transfer learning. APTOS 2019 was utilized for numerical experiments. AlexNet, Res-Net18, SqueezeNet, GoogleNet, VGG16, and VGG19 are used in this research. DenseNet and Inception-Resnet were chosen as the models of choice because of their higher layer counts. 

Model robustness and overfitting were both improved by the additional data. Moreover, Li et al. [35] created CANet to forecast DR utilizing ML models trained on Messidor and IDRiD challenge datasets, which predicted 85.10%. Image processing removed blood vessels, microaneurysms, and exudates by Afrin and Shill [36]. Measured blood vessel area, microaneurysm count, and exudate area from processed pictures and fed them into a knowledge-based fuzzy classifier for Classification, achieving 95.63% accuracy. Jena et al. [37] enhanced images using CLAHE on the green channel, and DR lesions were identified using a CNN coupled with a support vector machine (SVM). 

Based on the study’s outcomes on DR identification and diagnosis approaches, a significant number of gaps still require investigation. Due to the unavailability of a large amount of data, there has been little limitation on building and training a custom DL model entirely from scratch, reasoning from multiple studies that have attained outstanding trustworthiness values using pre-trained models with transfer learning. Furthermore, most of these experiments only trained DL models on raw photos, limiting the end classification network’s scalability. The new study incorporates multiple layers into the structure of pre-trained models to create a compact DR identification system, which solves these problems. As a result, the proposed system is more user-friendly and effective.

## 3. Approaches to Research

**Figure 2:**
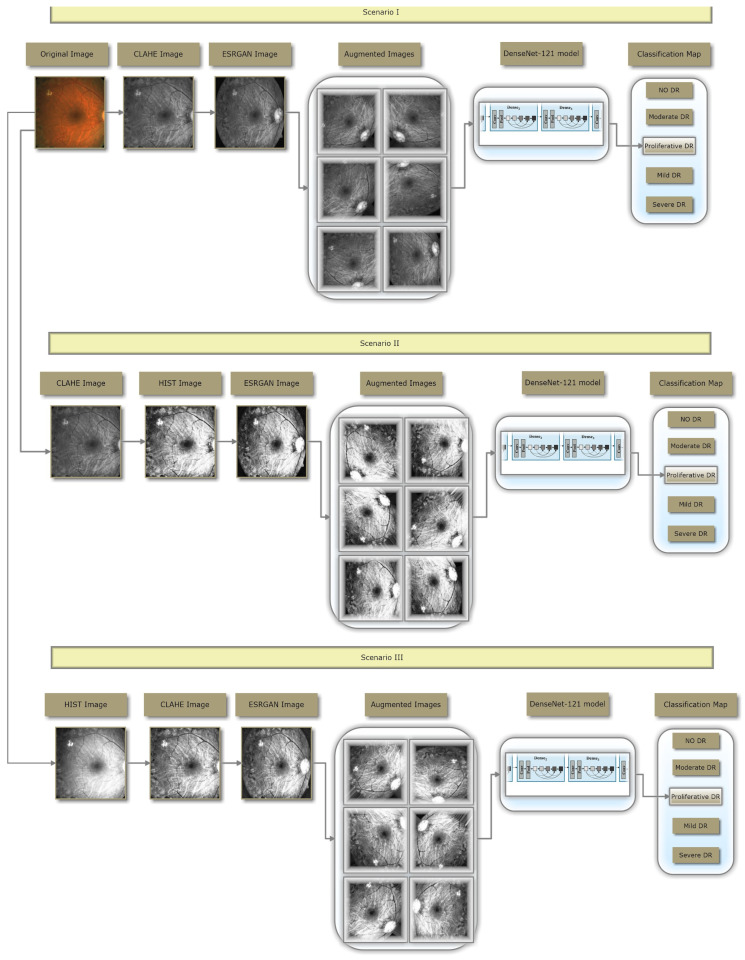
The process of DR classification.

As can be seen from Figure 2, a transfer DL approach (DenseNet-121) has been thoroughly trained within the image dataset to build racially discriminatory and useful feature representations for the DR detection system to operate. This section summarizes the strategy employed while processing the provided data. Next, the preprocessing stage is laid out in detail, and the implementation details of the proposed system are discussed; these include the three scenarios employed in this context, the techniques provided for preprocessing the data, a framework for the approach, and a way for training it.

### 3.1. Data Set Description

**Figure 3:**
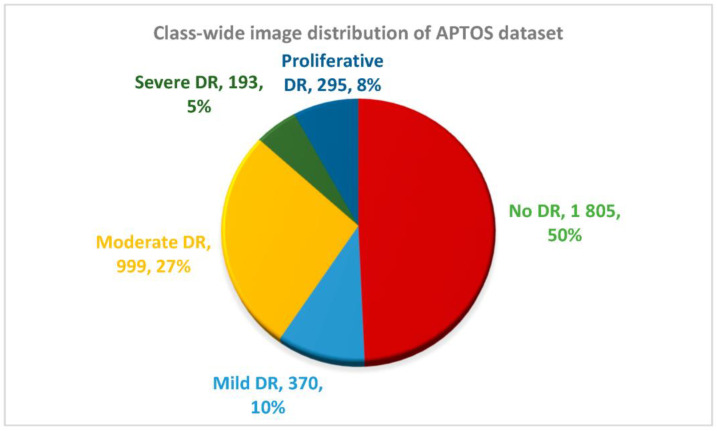
Class-Wide Image Distribution of the APTOS dataset.

When adopting a dataset, ensure there are enough high-quality images to operate on. The APTOS 2019 (Asia Pacific Tele-Ophthalmology Society) Blindness Detection Dataset [15] is employed for this research, one of many accessible Kaggle datasets, including thousands of images. The five stages of DR are represented here with high-resolution Retinal images, numbered from 0 (no DR) to 4 (proliferate DR), along with labels 1–4. There are 3662 images of the retina, with 193 in the severe DR group, 370 in the moderate DR group, 999 in the moderate to severe DR group, and 295 in the proliferate DR group (Figure 3). In Figure 1, we have seen several samples of the 3216 × 2136 pixel images. It ought to be expected that, as with any given dataset, there will be some random variation in both the images and the labels. The given photos may have artifacts, blurriness, poor brightness, and other problems. The images were taken by various individuals using various cameras at different clinics over a long period of time, all of which adds to the wide variety of the set as a whole.

### 3.2. Proposed Methodology

This article’s dataset was utilized to create an automatic DR classification model, and its workflow is presented in Figure 2. It shows three different scenarios: one in which preprocessing is carried out in two stages (using CLAHE and ESRGAN), the other two scenarios in which preprocessing is carried out in three stages (using CLAHE, HIST, and ESRGAN; and HIST, CLAHE, and ESRGAN for the second and third scenarios, respectively). The augmentation phase follows this phase to prevent overfitting. Eventually, the DenseNet-121model will be used to classify the images.

#### 3.2.1. An ESRGAN and CLAHE-Based Preprocessing

Retinal fundus images are frequently gathered from many sources using various methods. Consequently, given the considerable luminance variations in the photos used by the suggested protocol, it was crucial to enhance the quality of DR images and eliminate several sorts of noise. All photos in all scenarios are resized to a 224 × 224 × 3 resolution to best fit the inputs of the learning model. Since the brightness of each image’s pixels can vary widely, the data has been normalized between (−1) and (1) to maintain it within acceptable bounds and eliminate any noise. Normalizing the weights makes the model less susceptible to changes and therefore easier to tweak.

#### Scenario I

**Figure 4:**
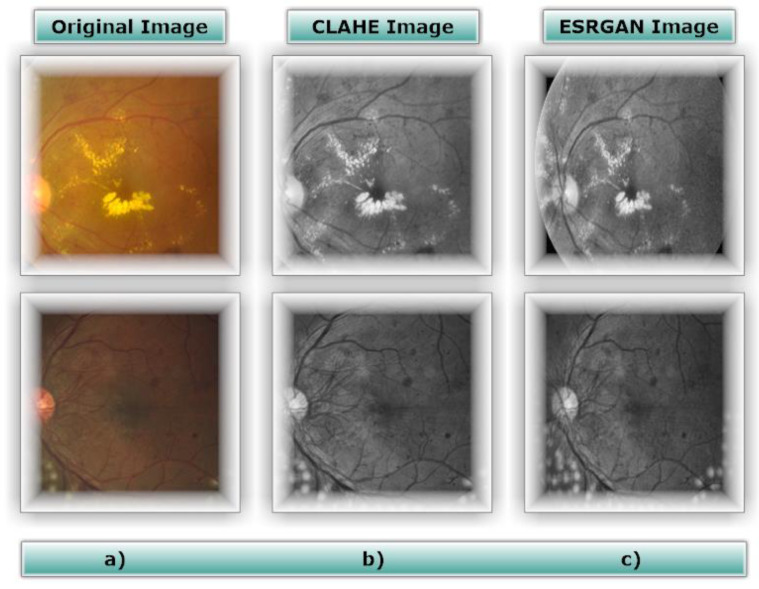
Various examples of the image-improvement methods that have been proposed (**a**) An unaltered version of the image; (**b**) a CLAHE version of the same image; and (**c**) an ESRGAN-enhanced version of the same image.

In scenario I, all images undergo an initial preprocessing phase before the augmentation and training phases. As can be seen in Figure 4b, CLAHE was first used to improve the DR image’s prominent features, patterns, and poor contrast by redistributing the input image’s luminance qualities [38]. To achieve this, the image was first segmented into many non-overlapping portions of about equal size. Therefore, the local luminance of an image is improved, while sharper edges and arcs are made more apparent by using this technique. Figure 4c shows the output from Stage 2 being sent into ESRGAN for further processing. When taking an ESRGAN photo, you can more accurately imitate the crisp edges that characterize image distortions [39]. Figure 4 shows one such strategy, which improves accuracy by increasing brightness while making the image’s edges and curves stand out more clearly.

#### Scenario II

**Figure 5:**
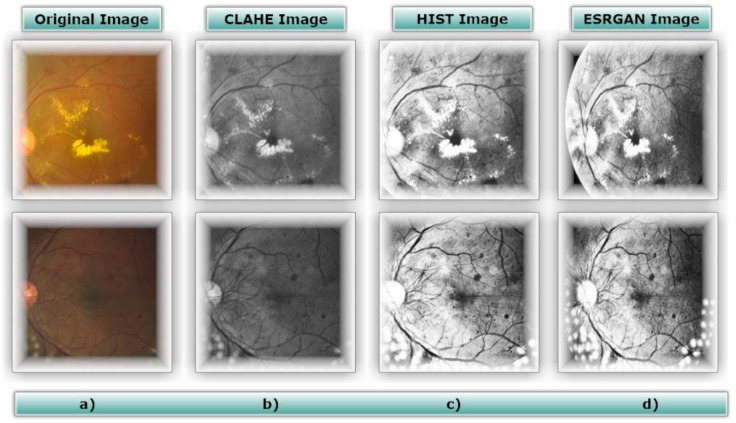
Some examples of the image-improvement methods that have been proposed. The four images shown here are: (**a**) the raw, unedited original; (**b**) the image after CLAHE; (**c**) the image utilizing HIST; and (**d**) the image after ESRGAN has been applied to it.

Like scenario I, all images in scenario II go through preliminary preprocessing before the augmentation and training stages. Figure 5b shows that the brightness attributes of the input image were redistributed using CLAHE to enhance the DR image’s salient features, patterns, and weak contrast. HIST [17,18] was applied to the output from stage 2, and the result is shown in Figure 5c. One definition of HIST is the distribution of a single data type. It is a method for enhancing an image’s contrast and overall visual quality. Equalizing the Histogram will expand the entire range of pixels from 0 to 255. Good contrast and discernible detail are hallmarks of a high-quality histogram. Finally, as shown in Figure 5d, ESRGAN is applied to the results of Stage 3. One such method is depicted in Figure 5; it enhances precision by brightening the image, bringing attention to its edges and curves.

#### Scenario III

**Figure 6:**
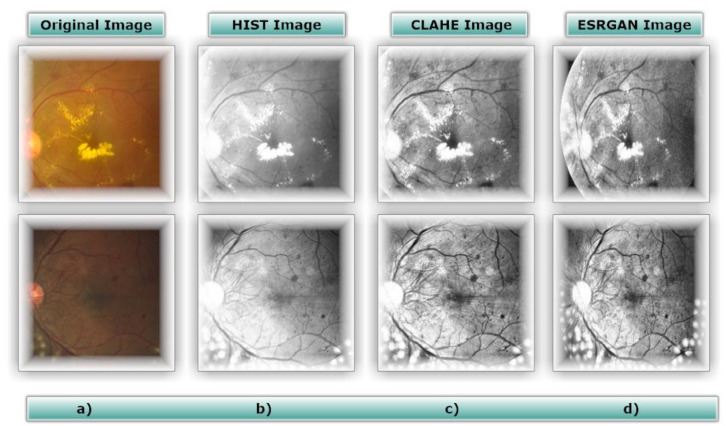
Some examples of image-improvement methods that have been proposed. The four images shown here are: (**a**) the raw, unedited original; (**b**) the image utilizing HIST; (**c**) the image after CLAHE; and (**d**) the image after ESRGAN has been applied to it.

Like scenario II, all images in scenario III go through preliminary preprocessing before the augmentation and training stages. Figure 6b shows that the brightness attributes of the input image were redistributed using HIST to enhance the DR image’s salient features, patterns, and weak contrast. After that, CLAHE was applied to the output from stage 2, and the result is shown in Figure 6c. Finally, as shown in Figure 6d, ESRGAN is applied to the results of stage 3. One such method is depicted in Figure 6; it enhances precision by brightening the image, bringing attention to the image’s edges and curves.

#### 3.2.2. Expansion of Data

**Figure 7:**
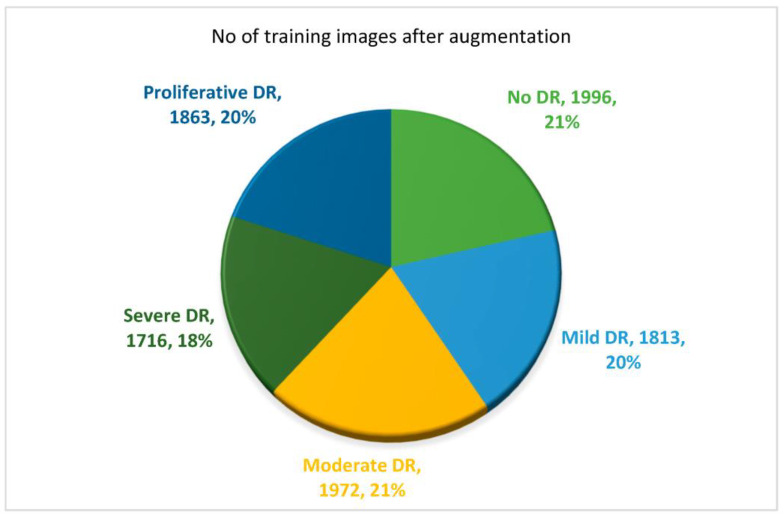
Total number of training images after augmentation techniques have been employed.

In order to introduce DenseNet-121 to a dataset with inconsistencies, researchers initially employed data augmentation to increase the number of images throughout the training sample. Once provided with more data to learn from, DL approaches generally improve their performance. We are able to make use of the special properties of DR imaging by tailoring our edits to each image. Scaling, inverting horizontally or vertically, and rotating the image a certain number of degrees do not affect the DNN’s precision. Overfitting is avoided, and the imbalance in the dataset is corrected by the application of data augmentations (i.e., translation, rotation, and magnification). Horizontal shift augmentation is one of the transformations considered for this study; it involves horizontally shifting an image’s pixels while maintaining the original image’s perspective. The dimension of this transition is specified by a number ranging from 0 to 1, and the viewing angle of the original image is preserved. The image can also be rotated, an additional type of transformation, by a random amount between 0 and 180 degrees. By employing data augmentation methods, we were able to fix the problem of varying sample sizes and convoluted categorizations. The APTOS dataset is a good example of an “imbalanced class”, defined as an uneven distribution of samples across various classes, as shown in Figure 3. Figure 7 illustrates how the dataset’s classes are evenly distributed throughout all scenarios after applying augmentation techniques. 

**Figure 8:**
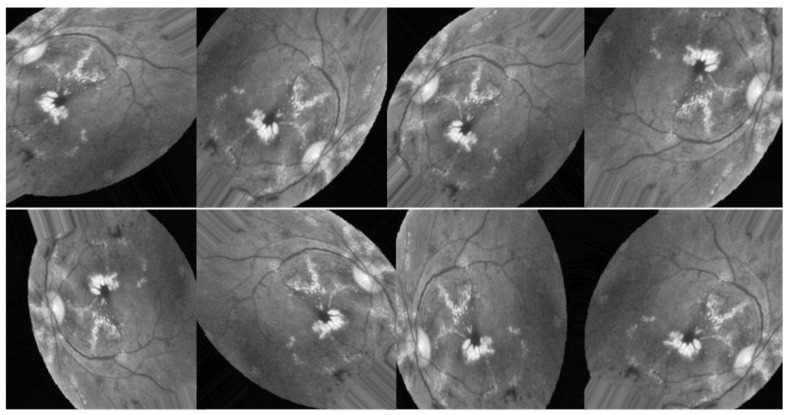
Examples of augmenting the same image with different methods (CLAHE + ESRGAN).

**Figure 9:**
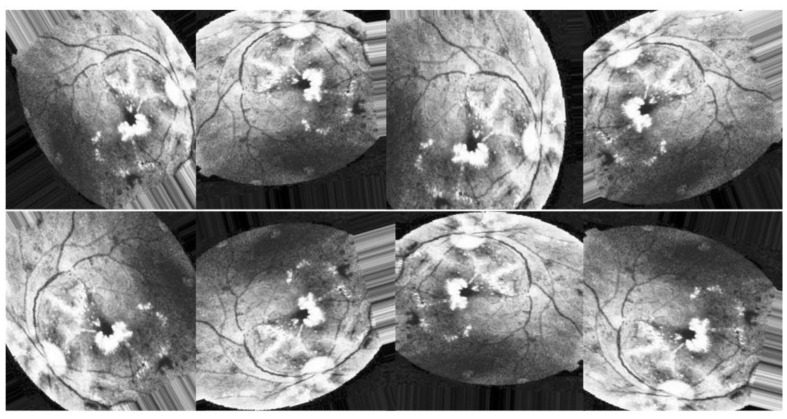
Examples of augmenting the same image with different methods (CLAHE + HIST + ESRGAN).

**Figure 10:**
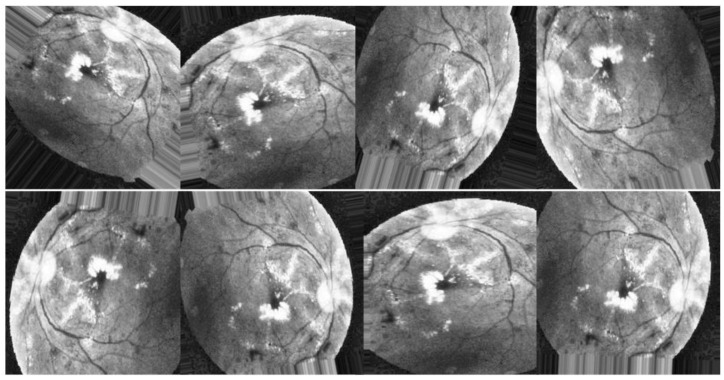
Examples of augmenting the same image with different methods (HIST + CLAHE + ESRGAN).

All previous edits to images in the training set are applied to generate new samples for the network. While the total number of images is the same in all scenarios, Figure 8, Figure 9 and Figure 10 illustrate the purpose of data augmentation, which is to increase the quantity of data by providing slightly altered copies of the existing data or newly synthesized data derived from the existing data using the same parameters in all three scenarios. Here are the three scenarios that were used to train DenseNet-121:

#### Scenario I

In the first scenario, shown in Figure 8, researchers augment the improved images using CLAHE and ESRGAN.

#### Scenario II

The second scenario is to apply augmentation techniques to the enhanced images utilizing CLAHE, HIST, and ESRGAN, respectively, as depicted in Figure 9.

#### Scenario III

Finally, in the third scenario, augmentation techniques are applied to the enhanced images utilizing HIST, CLAHE, and ESRGAN, respectively, as depicted in Figure 10. 

#### 3.2.3. Learning Model (DenseNet-121)

The Dense Convolutional Network (DCN) is a type of network infrastructure in which every layer is deeply related to every other layer. Most of the other layers’ feature maps are viewed as independent input variables for each layer, while their own feature maps are passed on to all of the layers that come after them [16]. DenseNets are better than other DCNs because they address the issue of vanishing gradients, improve feature spreading, motivate feature reuse, and minimize the number of parameters by a large amount. Most of the time, DenseNets function better than the state-of-the-art while consuming less memory and computation [16].

## 4. Experimental Results

### 4.1. Criteria for Assessment

This part details the methods used to assess the study’s success and its final results. Classifier Accuracy is a popular metric for gauging classification performance. By dividing the total number of examples by the percentage of valid identifications, we arrive at the formula shown in Equation (1). Image categorization performance is typically evaluated based on metrics like sensitivity and specificity. The accuracy of the specificity formula presented by Equation (2) improves as more images are correctly labeled. Using Equation (3), we counted how many images in the dataset exhibited a linear correlation. A higher F-score indicates that the system is more likely to make correct predictions. The value of a system cannot be gauged solely by its accuracy and sensitivity. Equation (4) provides the formula for computing the F-score (Fsc). Fourthly, we looked at how well the model N’s highest likelihood responses followed the expected softmax distribution (also known as the “top N accuracy”). The effectiveness of the classification is determined by whether or not one of the N predictions corresponds to the actual label.

(1) $$Accuracy=\frac{true\_positive+true\_negative}{true\_positive+true\_negative+flase\_positive+flase\_negative}$$

(2) $$Specificity=Precision=\frac{true\_negative}{true\_negative+flase\_positive}$$

(3) $$Sensitivity=Recall=\frac{true+positive}{true\_positive+flase\_negative}$$

(4) $$F1-Score=2*\left(\frac{Precision*Recall}{Precision+Recall}\right)$$

### 4.2. Instruction and Setup of DenseNet-121

The APTOS dataset validated the DL system and compared its performance to best practices. According to the preferred training strategy, 80% was used for training (9360 photos) and 10% for testing (549 images). Moreover, 549 photos, or 10%, were randomly selected to serve as a validation set for assessing performance and retaining optimal weight combinations. Images were reduced in size during training to a 224 × 224 × 3-pixel resolution. A Linux PC with an RTX3060 GPU and 8 GB of RAM tested the proposed system’s TensorFlow Keras application. The suggested system utilizes the Adam optimizer and a learning rate approach that delays the learning rate.

In contrast, learning has stagnated for a long time and has been pre-trained on the APTOS dataset (i.e., validation patience). Adam optimized these training hyperparameters: The simulation runs for 50 epochs with a learning rate between 1 × 10^3^ and 1 × 10^5^, a batch size between 2 and 64, a 2× increment, 10 patience steps, and 0.90 momentum. To complete our variety of anti-infective approaches, we apply a “batching” technique for dispersing diseased species. 

### 4.3. Observations on the DenseNet-121Model’s Efficacy

**Figure 11:**
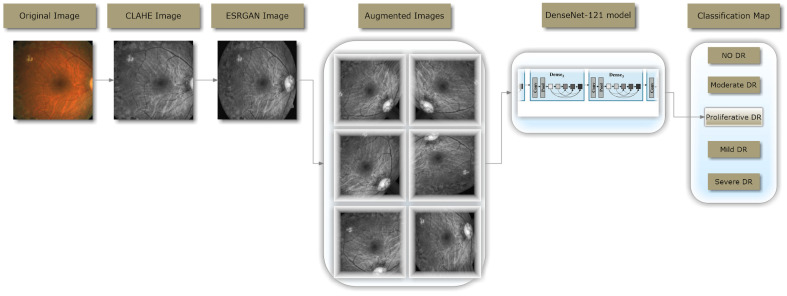
Scenario I-specific workflow depiction of the DR detection system.

Figure 11 depicts the results of an evaluation of three different instance sets for the APTOS dataset, where DenseNet-121 was applied to the dataset in three different enhancement scenarios: (a) CLAHE + ESRGAN, (b) CLAHE + HIST + ESRGAN, and (c) HIST + CLAHE + ESRGAN. Each data set is split into 80% training, 10% validation, and 10% testing samples. This division was implemented to reduce the overall duration of the project. The model is trained for 50 epochs using 2, 4, 8, 32, and 64 as batch sizes and 1 × 10^3^, 1 × 10^4^, and 1 × 10^5^ as learning rates. To ensure the utmost accuracy, DensNet-121 has been fine-tuned by freezing between 140 and 160 layers. Model ensembles are constructed by repeatedly executing the same model with the same parameters, and since performance varies from run to run because of the random weights established for each run, only the best run result is recorded and supplied. The optimal outcomes for each Scenario, as calculated by the DenseNet-121 model, are detailed below.

#### Scenario I

The first scenario used is depicted in Figure 11. In this scenario, preprocessing is conducted in two steps (utilizing CLAHE and ESRGAN), and then augmentation is used to prevent overfitting. The images are ultimately classified using the DensNet-121 model. Table 1 demonstrates that scenario I yields the highest performance when used, with an accuracy of 98.36 percent, a top-2 accuracy of 100 percent, a top-3 accuracy of 100 percent, a precision of 98 percent, a recall of 98 percent, and an F1-score of 98 percent. The APTOS dataset shows the total number of images tested across all categories in Table 2. As can be seen from the data, the No DR class has the most instances (270) and the highest Precision, Recall, and F1-score values (100, 99, and 99, respectively).

**Figure 12:**
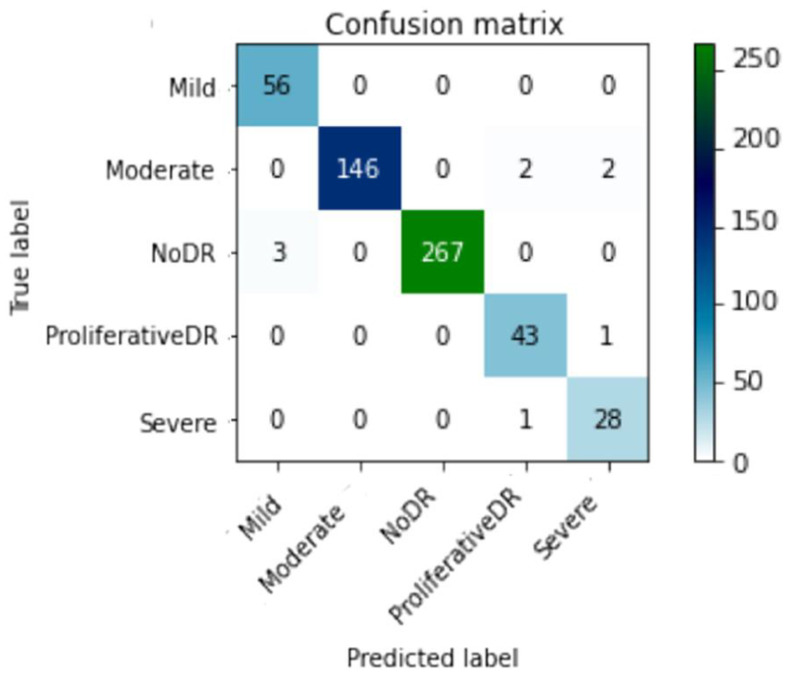
The finest DenseNet-121 confusion matrix with enhancement (CLAHE + ESRGAN).

An evaluation of a classification model’s accuracy on a validation set is shown in Figure 12 through a comparison of the actual and predicted labels. We tested our model using a single-label classification approach for five classes, and the results are depicted in Figure 12 below as the confusion matrix. The confusion matrix displays the discrepancy between the true and predicted labels for each image in the validation set. Components on the diagonal represent the fraction of instances where the classifier correctly predicted the label, whereas non-diagonal elements represent instances where the classifier made a mistake. 

#### Scenario II

**Figure 13:**
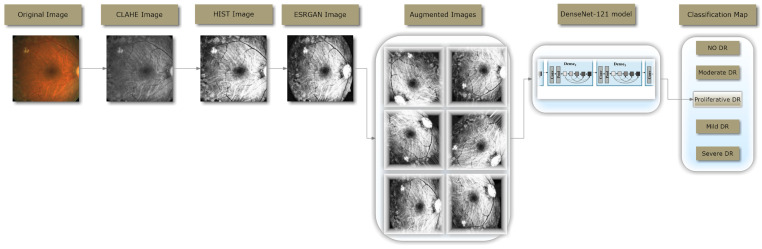
Scenario II-specific workflow depiction of the DR detection system.

The second scenario is depicted in Figure 13. In this scenario, preprocessing is conducted in three steps (utilizing CLAHE, HIST, and ESRGAN), and then augmentation is used to prevent overfitting. The images are ultimately classified using the DenseNet-121 model. 

**Figure 14:**
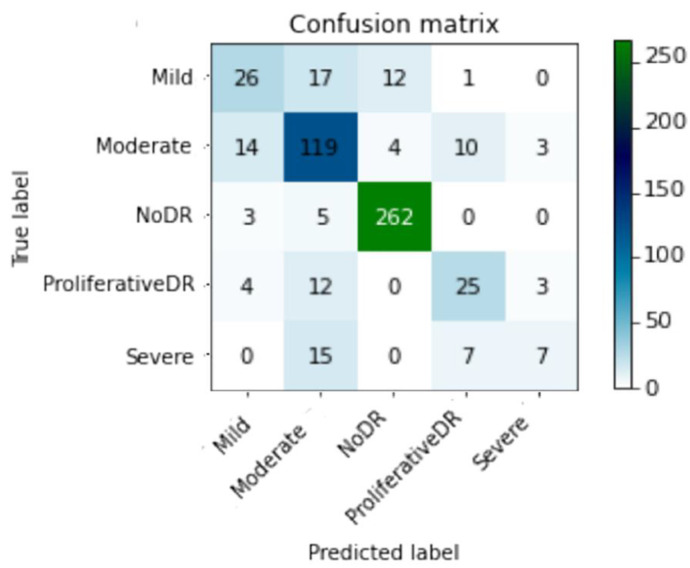
The finest DenseNet-121 confusion matrix with enhancement (CLAHE + HIST + ESRGAN).

Table 3 demonstrates that scenario II yields the highest performance when used, with an accuracy of 79.96 percent, a top-2 accuracy of 89.62 percent, a top-3 accuracy of 97.09 percent, a precision of 79 percent, a recall of 80 percent, and an F1-score of 79 percent. The APTOS dataset shows the total number of images tested across all categories in Table 4. As can be seen from the data, the No DR class has the most instances (270) and the highest Precision, Recall, and F1-score values (94, 97, and 96, respectively). Figure 14 reveals the best confusion matrix of DenseNet-121 for scenario II.

#### Scenario III

**Figure 15:**
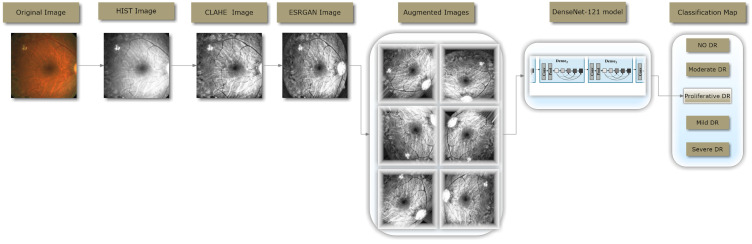
Scenario III-specific workflow depiction of the DR detection system’.

**Figure 16:**
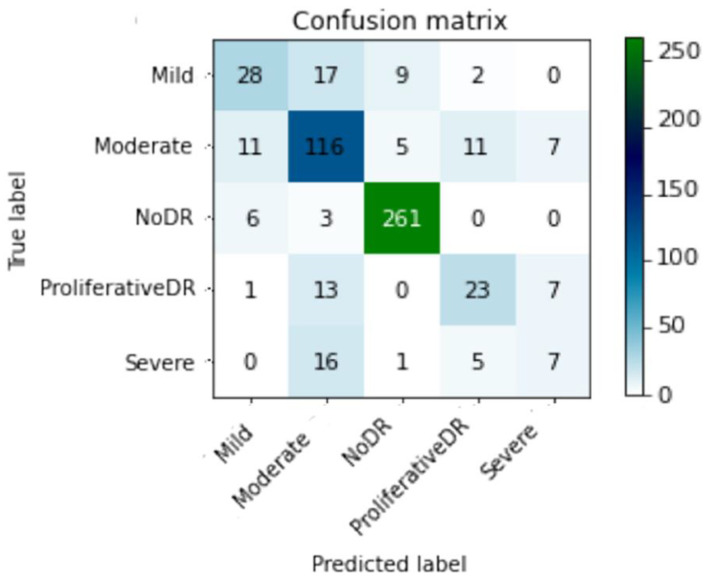
The finest DenseNet-121 confusion with enhancement (HIST + CLAHE + ESRGAN).

The third scenario used is depicted in Figure 15; in this scenario, preprocessing is conducted in three steps (utilizing HIST, CLAHE, and ESRGAN), and then augmentation is used to prevent overfitting. The images are ultimately classified using the DenseNet-121 model. Table 5 demonstrates that scenario III yields the highest performance when used, with an accuracy of 79.23 percent, a top-2 accuracy of 90.35 percent, a top-3 accuracy of 96.72 percent, a precision of 78 percent, a recall of 79 percent, and an F1-score of 79 percent. The APTOS dataset shows the total number of images tested across all categories in Table 6. As can be seen from the data, the No DR class has the most instances (270) and the highest Precision, Recall, and F1-score values (95, 97, and 96, respectively). Figure 16 reveals the best confusion matrix of DenseNet-121 for scenario III.

### 4.4. Contrast and Comparison of the Various Methodologies

**Figure 17:**
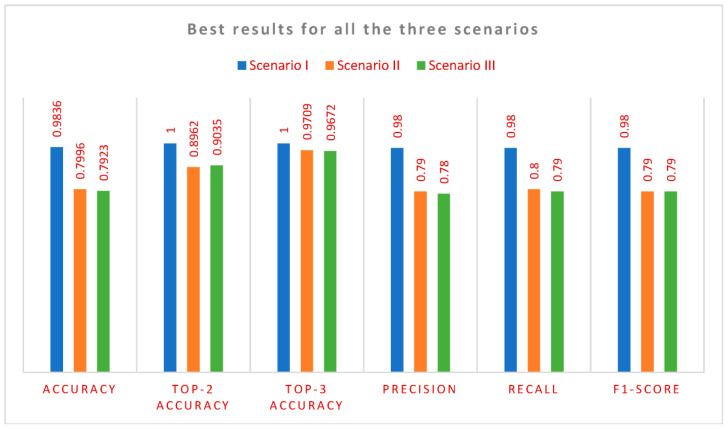
Best results for the three scenarios.

**Figure 18:**
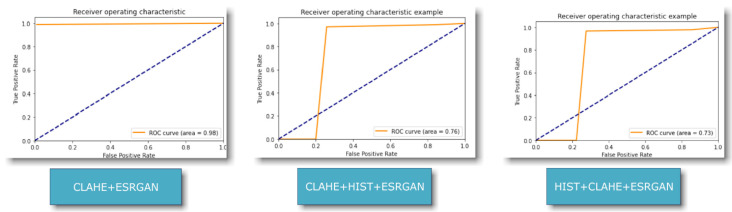
ROC curve for the three scenarios.

According to the assessment measures used, scenario I with CLAHE and ESRGAN yields the best result compared to the other offered scenarios, as depicted in Figure 17. Diagnostic efficacy was determined by calculating the area under the receiver operating characteristic (ROC) curve, which depicts a given model’s true positive and false positive rates. The area under the ROC curve (AUC) can be calculated by adding the areas of the individual trapezoidal pieces. Figure 18 displays the AUC assessments for the three scenarios using the proposed technique. The AUC is likewise comparable across all figures, as shown in Figure 18. With an AUC of 0.98, the first scenario provided performs marginally better than the others.

### 4.5. Evaluating Several Alternative Approaches

As seen in Table 7, our approach is superior to other methods in terms of both efficacy and performance. Its efficacy is weighed against that of similar approaches. Compared to the top existing approaches, the proposed inception model exhibits an efficiency rating of 96.36% regarding scenario I.

### 4.6. Discussion

The authors developed a new classification system for DR incorporating CLAHE, HIST, and ESRGAN aspects. The created model was tested on the DR photos from the APTOS 2019 dataset. Thus, the APTOS dataset is employed in three different scenarios: Scenario I, which involves CLAHE and ESRGAN; scenario II, which involves CLAHE + HIST and ESRGAN; and scenario III, which involves HIST + CLAHE and ESRGAN. The model achieved a 98.36 percent accuracy across five classes in scenario I of the 80:20 hold-out validation and a 79.96% and 79.23% accuracy across scenarios II and III, respectively. For classification in all cases where the proposed method was used, a pre-trained DenseNet-121 architecture has been used. 

Our experiments show that the DenseNet architecture offers several substantial benefits over the alternatives. At the outset, the authors boast that their design outperforms the competition in ImageNet. Our Near-Identical Image analysis confirms this, showing that the DenseNet architecture yields the most accurate depiction of pictures. Second, the authors state that their technique makes it simpler to train the network due to increased parameter efficiency. This is true when compared to other similarly sized network configurations. Our argument is that the training time is comparable to that of some lower-layer networks. The benefits of the additional training time are undeniable. 

In light of the promising results obtained by our previous work “DL-Based Prediction of DRUsing CLAHE and ESRGAN for Enhancement” on the same dataset (APTOS) using a different DL model (Inception-V3), additional work has been performed using Histogram equalization to test its effect in consequence with CLAHE and ESRGAN.

Throughout model development, we compared the categorization performances of three distinct scenarios and found that scenario III’s enhancement strategy yielded the best overall results achieved through the use of augmentation methods employed in Scenario I (Figure 17). As can be seen in Table 7, the outcomes of Scenarios II and III are weaker than those of scenario I but are still competitive with other studies ([46,49,50] utilizing the VGG-16 model). We provide empirical proof that the general resolution increase of CLAHE + ESRGAN is the key contributor to our methodology’s significant accuracy gains. The relatively small size of the sample and the requirement that all images in the dataset have approximately the same resolution are the study’s main limitations. In order to draw reliable findings from a study, it is vital to have a significant sample size. The larger the sample, the more accurate the results; hence, more samples are required to improve the testing result.

Table 8 shows the proposed model’s performance under various enhancement situations; the results demonstrate that the model learns well without overfitting, as the difference between the three sets of predictions is small.

**Figure 19:**
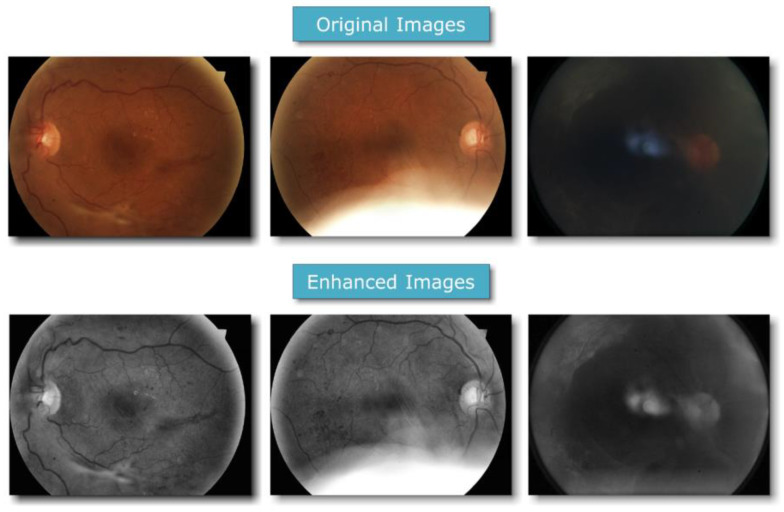
Original and enhanced image samples.

Figure 19 illustrates a sample of photographs belonging to the same class, demonstrating that applying the suggested improvement strategy to the EyePACS dataset provided poor results due to the wide variety of the acquired images and their poor quality. Despite the best improvement approach proposed (CLAHE + ESRGAN), the image quality still fluctuates from one image to the next depending on the nature and resolution of the original image.

**Figure 20:**
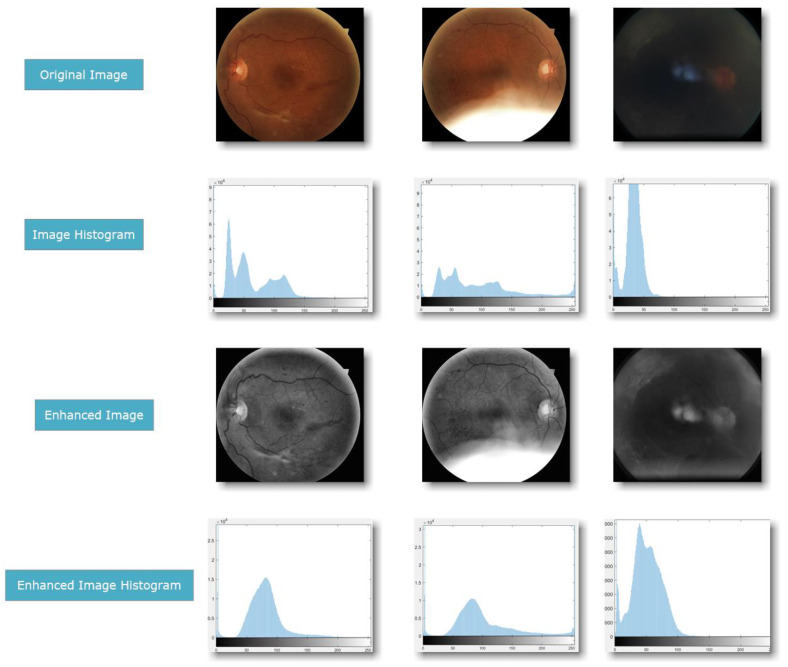
Original and Enhanced Images + Histogram.

The histogram of images from the moderate DR class before and after CLAHE + ESRGAN processing is shown in Figure 20. The image is first converted to grayscale, then the intensity of each pixel is normalized throughout the full Histogram using CLAHE, and finally the image is sharpened using ESRGAN.

**Figure 21:**
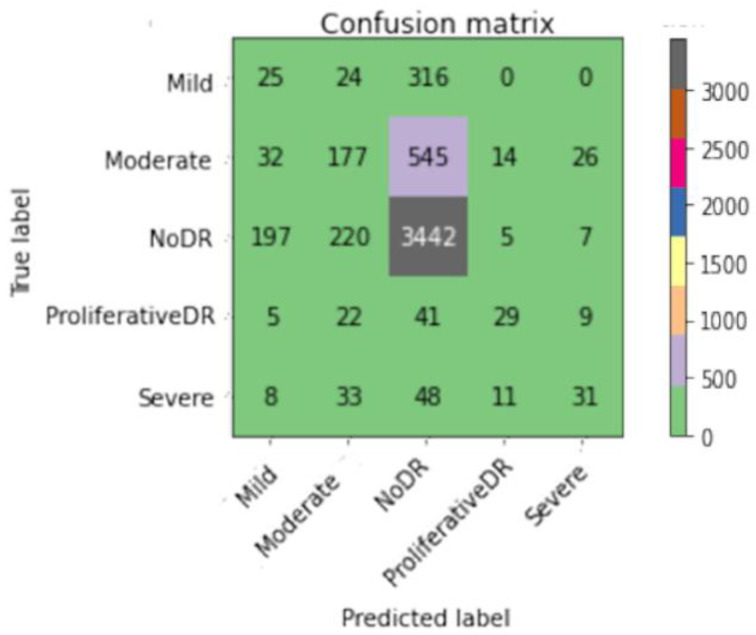
Superior Confusion Matrix for the EyePACS dataset.

**Figure 22:**
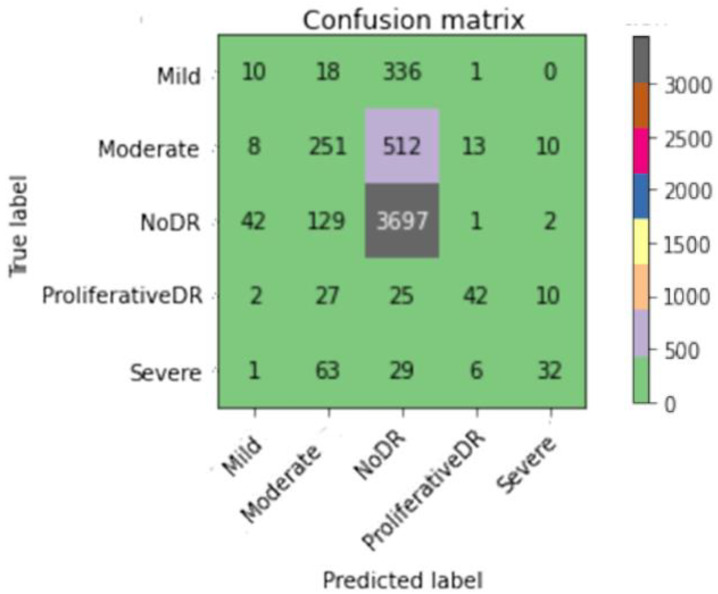
Superior Confusion Matrix for the retrained APTOS model using the EyePACS dataset.

Figure 21 shows that the testing accuracy is increased by 70.32 percent when CLAHE + ESRGAN is employed as a preprocessing step on images from the EyePACS dataset. EyePacs has undergone further testing, with positive results (76.55%) achieved through retraining the taught model with APTOS, as shown in Figure 22. 

When all images in a dataset have roughly the same resolution, we discovered that the high accuracy improvements achieved by our technique are primarily attributable to the overall resolution enhancement provided by CLAHE + ESRGAN. When compared to alternative scenarios, the time required is drastically reduced when CLAHE + ESRGAN is used as the improvement step. The study’s findings back up these anecdotes.

## 5. Conclusions

The APTOS collection contains retinal images, and researchers have developed a method for rapidly and precisely evaluating five different types of cancer. Three scenarios are used in the suggested method: Scenario I utilizes CLAHE and ESRGAN, scenario II utilizes CLAHE, HIST, and ESRGAN; and scenario III utilizes HIST, CLAHE, and ESRGAN. DenseNet-121 is trained on the leading edge of preprocessed medical imaging, employing augmentation approaches to avoid overfitting and enhance the suggested methodology’s overall capabilities. The approach claims that when using DenseNet-121, the conception model has a prediction performance comparable to that of trained ophthalmologists: 98.36%, 79.96%, and 79.23% for scenarios I, II, and III, respectively. In addition to applying different augmentation methods, each with its own set of parameters, to generate a wide range of visually distinct samples, the research’s novelty and relevance stem from the use of CLAHE and ESRGAN in the preprocessing phase, which differs from our previous work by expanding the results by applying more scenarios (CLAHE + HIST + ESRGAN and HIST + CLAHE + ESRGAN). The study uses the APTOS dataset to demonstrate that the suggested strategy outperforms state-of-the-art methods. Testing on a huge and complicated dataset, including plenty of future DR instances, must be conducted to prove the recommended technique’s effectiveness. Future analyses of fresh datasets could use augmentation techniques like AlexNet, EfficientNet, or Inception-ResNet. Additionally, new enhancement methods could improve the image’s quality.

## Figures and Tables

**Table 1:** Superior accuracy via improvement (CLAHE + ESRGAN).

Acc	Top-2 Accuracy	Top-3 Accuracy	Precision	Recall	F1-Score
0.9836	1.00	1.00	0.98	0.98	0.98

**Table 2:** Detailed results for each class using CLAHE + ESRGAN.

	Precision	Recall	F1-Score	Total Images
Stage 0	1.00	0.99	0.99	270
Stage 1	1.00	0.97	0.99	150
Stage 2	0.95	1.00	0.97	56
Stage 3	0.90	0.97	0.93	29
Stage 4	0.93	0.98	0.96	44
Average	0.98	0.98	0.98	549

**Table 3:** Superior Accuracy via Improvement (CLAHE +HIST + ESRGAN).

Acc	Top-2 Accuracy	Top-3 Accuracy	Precision	Recall	F1-Score
0.7996	0.8962	0.9709	0.79	0.80	0.79

**Table 4:** Detailed results for each class using CLAHE + HIST + ESRGAN.

	Precision	Recall	F1-Score	Total Images
Stage 0	0.94	0.97	0.96	270
Stage 1	0.71	0.79	0.75	150
Stage 2	0.55	0.46	0.50	56
Stage 3	0.54	0.24	0.33	29
Stage 4	0.58	0.57	0.57	44
Average	0.79	0.80	0.79	549

**Table 5:** Superior Accuracy via Improvement (HIST + CLAHE + ESRGAN).

Acc	Top-2 Accuracy	Top-3 Accuracy	Precision	Recall	F1-Score
0.7923	0.9035	0.9672	0.78	0.79	0.79

**Table 6:** Detailed results for each class using HIST + CLAHE + ESRGAN.

	Precision	Recall	F1-Score	Total Images
Stage 0	0.95	0.97	0.96	270
Stage 1	0.70	0.77	0.74	150
Stage 2	0.61	0.50	0.55	56
Stage 3	0.33	0.24	0.28	29
Stage 4	0.56	0.52	0.54	44
Average	0.78	0.79	0.79	549

**Table 7:** Comparison of system performance to previous research using the APTOS dataset.

Reference	Technique	Accuracy
[2]	MSA-Net	84.6%
[40]	EfficientNet-B6	86.03%
[41]	SVM	94.5%
[42]	SVM classifier and MobileNet_V2 for feature extraction	88.80%
[43]	Densenet-121, Xception, Inception-v3, Resnet-50	85.28%
[26]	Inception-ResNet-v2	72.33%
[44]	MobileNet_V2	93.09%
[45]	EfficientNet and DenseNet	96.32%
[46]	VGG16	96.86%
[47]	CNN	95.3%
[48]	Hybrid Residual U-Net	94%
[49]	Inception-ResNet-v2	97.0%
[50]	VGG-16	74.58%
[51]	VGG16	73.26%
	DenseNet121	96.11%
[52]	LBCNN	97.41%
[53]	Inception-v3	88.1%
[54]	DenseNet201	93.85%
Proposed Methodology	DenseNet-121(using CLAHE + ESRGAN) scenario I	98.36%
	DenseNet-121(using CLAHE + HIST + ESRGAN) scenario II	79.96%
	DenseNet-121(using HIST + CLAHE + ESRGAN) scenario III	79.23%

**Table 8:** Examination of the accuracy of the model throughout training, validation, and testing.

Scenario	Enhancement Technique	Training Accuracy	Validation Accuracy	Testing Accuracy
I	CLAHE + ESRGAN	0.9858	0.9709	0.9836
II	CLAHE + HIST + ESRGAN	0.8216	0.7978	0.7996
III	HIST + CLAHE + ESRGAN	0.8362	0.8069	0.7923

## Data Availability

Will be furnished on request.
